# Supplementary material for: Acoustic-driven magnetic skyrmion motion
Source: Nat Commun. 2024 Feb 3;15:1018. doi: 10.1038/s41467-024-45316-w (PMC10838300; doi:10.1038/s41467-024-45316-w)
Supplement: Supplementary file 1 — Supplementary Information [file 41467_2024_45316_MOESM1_ESM.pdf]

Supplementary Information

Acoustic-Driven Magnetic Skyrmion Motion

Yang Yang[1#], Le Zhao[2#], Di Yi[3#], Teng Xu[2], Yahong Chai[1], Chenye Zhang[1], Dingsong Jiang[1], Yahui Ji[1], Dazhi Hou[4,5], Wanjun Jiang[2*], Jianshi Tang[1], Pu Yu[2], Huaqiang Wu[1], Tianxiang Nan[1*]

[1] School of Integrated Circuits and Beijing National Research Center for Information Science and Technology (BNRist), Tsinghua University, Beijing, China.

[2] Department of Physics, Tsinghua University, Beijing, China.

[3] School of Materials Science and Engineering, Tsinghua University, Beijing, China.

[4] ICQD, Hefei National Laboratory for Physical Sciences at Microscale, University of Science and Technology of China, Hefei, Anhui, China.

[5] Department of Physics, University of Science and Technology of China, Hefei, Anhui, China.

[#] These authors contributed equally.

[*] Corresponding author, e-mail: jiang_lab@tsinghua.edu.cn; nantianxiang@mail.tsinghua.edu.cn

I. Characterization of the devices

Fig. S1a shows the out-of-plane magnetic hysteresis loop of the sample characterized by a polar magneto-optic Kerr effect (MOKE) microscope. Upon applying an in-plane magnetic field pulse ($\mathbf{H}_{ip}$ = 30 mT) with a zero out-of-plane magnetic field, the maze domain transforms into Néel-type magnetic skyrmions (Fig. S1b). We fit an individual skyrmion profile by a Gaussian fitting (Fig. S1c). The shear horizontal (SH) wave is a leaky surface acoustic wave (SAW) for the free surface of the 64°Y-cut $LiNbO_3$ substrate. The use of heavy metal interdigital electrodes allows acoustic energy to concentrate on the surface and increases SH wave amplitude. The SH-type leaky SAW is confined on the surface using interdigital transducers (IDTs) consisting of heavy metal Pt electrodes on top of the $LiNbO_3$ substrate. The optical microscopic image of the sample is shown in Fig. S1d.

a

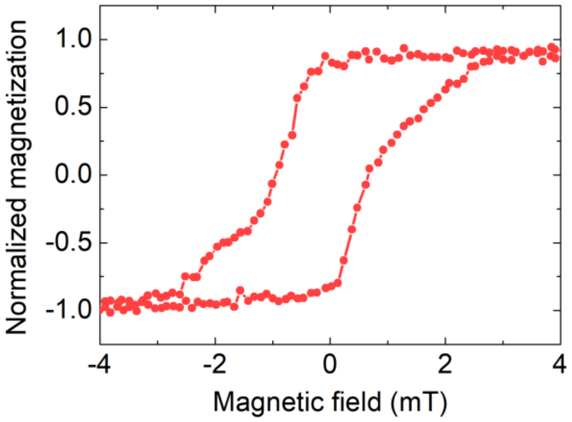

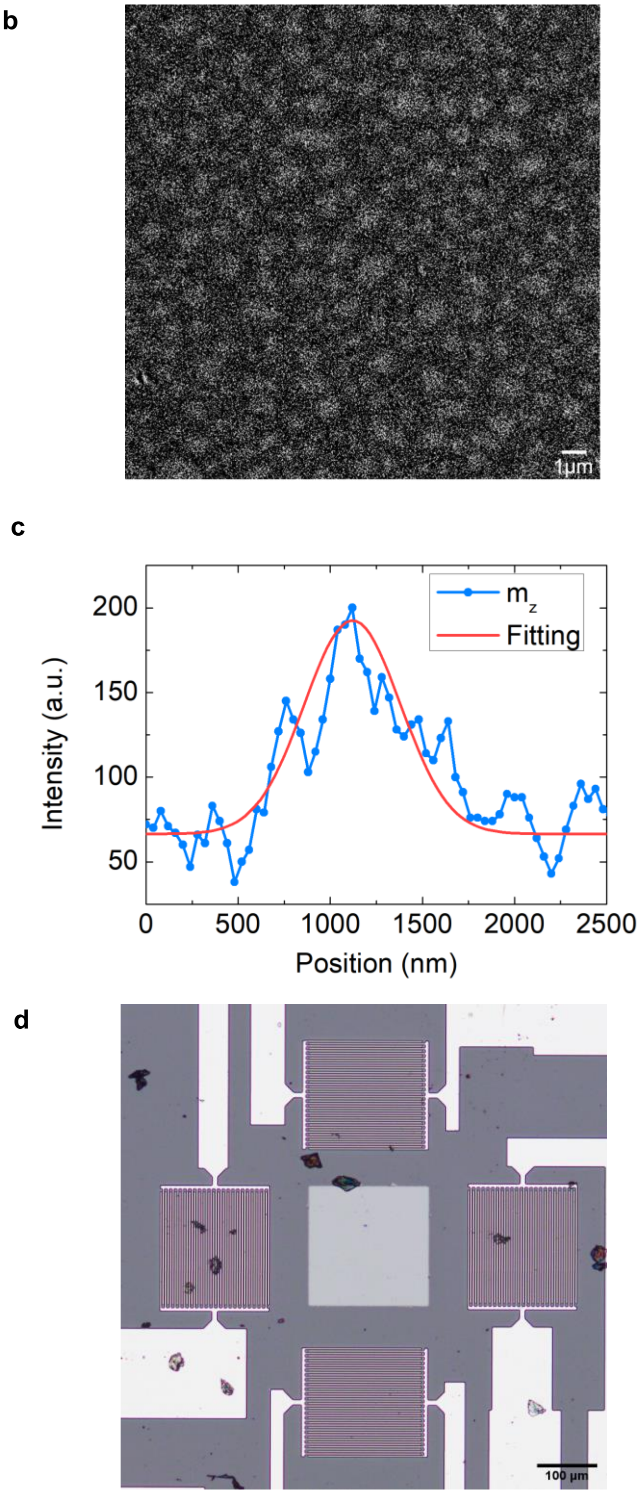

Supplementary Fig. S1 | The magnetic hysteresis loop, skyrmion image, the diameter of a skyrmion, and the optical microscopic image. a, The measured out-of-plane magnetic hysteresis loop of Ta/$Co_{20}Fe_{60}B_{20}$/MgO/Ta by a polar magneto-optic Kerr effect (MOKE) microscope. **b**, The MOKE image of the magnetic skyrmions after applying an in-plane magnetic field pulse with a zero out-of-plane magnetic field. **c**, A Gaussian fitting of an individual skyrmion profile. **d,** The optical microscopic image of the sample device.

II. Simulation methods

The micromagnetic simulation is implemented by solving the Landau–Lifshitz–Gilbert equation including interfacial Dzyaloshinskii-Moriya interaction, exchange interaction, magnetic anisotropy, magnetostatic, and magnetoelastic coupling contributions using MuMax3[1-3]. The whole code and code comments for an SH wave are as follows

```
d := 30
Setgridsize(256, 256, 2)
Setcellsize(1e-9, 1e-9, 0.5e-9)    // Define the geometry and meshes
Msat = 580e3                        // Define a saturation magnetization
Aex = 10e-12                        // Define an exchange constant
alpha = 0.1                         // Define a damping parameter
Dind = 3e-3                         // Define an interfacial Dzyaloshinskii-Moriya constant
anisu = vector(0, 0, -1)            // Define the uniaxial anisotropy direction
Ku1 = 7e5                           // Define the first order uniaxial anisotropy constant
Ku2 = 1.5e5                         // Define the second order uniaxial anisotropy constant
C11 = 283e9                         // Define elastic constants
C12 = 166e9
C44 = 58e9
rho = 8e3
B1 = -8.8e6                   // Define the first order magnetoelastic coupling constant
B2 = -8.8e6                   // Define the second order magnetoelastic coupling constant
Temp = 0                      // Temperature
m = Uniform(0, 0, -1)         // Initial magnetization state
m.setInShape(circle(d*1e-9).transl(-50e-9, 0, 0), NeelSkyrmion(-1, 1).scale(1,1, 1).transl(-50e-9, 0, 0))
// define the skyrmion size, type and position
relax()                             // Minimize the total energy
for i:=0; i<256; i++{
defregion(i,xrange(-127e-9+i*1e-9,-126e-9+i*1e-9))
frozenDispLoc.SetRegion(i,1)
frozenDispVal.SetRegion(i,vector(0,1e-9*sin(2*pi/0.24e-6*i*1e-9-2*pi*486e6*t),0))
// Define an SH wave
}
SetSolver(9)                        // Solver with magnetoelastic interaction
fixdt = 1e-14                       // Set a fixed time step
autosave(m, 1e-9)                   // Magnetization output
autosave(Edens_mel, 1e-9)           // Magnetoelastic energy density output
autosave(Edens_total, 1e-9)         // Total energy density output
run(400e-9)                         // Running
```

The whole code and code comments for a Rayleigh wave are as follows

```
d := 30
Setgridsize(256, 256, 2)
```

```
Setcellsize(1e-9, 1e-9, 0.5e-9)    // Define the geometry and meshes
Msat = 580e3                        // Define a saturation magnetization
Aex = 10e-12                        // Define an exchange constant
alpha = 0.1                         // Define a damping parameter
Dind = 3e-3                         // Define an interfacial Dzyaloshinskii-Moriya constant
anisu = vector(0, 0, -1)            // Define the uniaxial anisotropy direction
Ku1 = 7e5                           // Define the first order uniaxial anisotropy constant
Ku2 = 1.5e5                         // Define the second order uniaxial anisotropy constant
C11 = 283e9                         // Define elastic constants
C12 = 166e9
C44 = 58e9
rho = 8e3
B1 = -8.8e6                         // Define the first order magnetoelastic coupling constant
B2 = -8.8e6                         // Define the second order magnetoelastic coupling constant
Temp = 0                            // Temperature
m = Uniform(0, 0, -1)               // Initial magnetization state
m.setInShape(circle(d*1e-9).transl(-50e-9, 0, 0), NeelSkyrmion(-1, 1).scale(1,1, 1).transl(-50e-9, 0, 0))
// define the skyrmion size, type and position
relax()                             // Minimize the total energy
for i:=0; i<256; i++{
defregion(i,xrange(-127e-9+i*1e-9,-126e-9+i*1e-9))
frozenDispLoc.SetRegion(i,1)
frozenDispVal.SetRegion(i,vector(-1e-9*2/3*cos(2*pi/0.24e-6*i*1e-9-2*pi*451e6*t),0,1e-
9*sin(2*pi/0.24e-6*i*1e-9-2*pi*451e6*t)))
// Define a Rayleigh wave
}
SetSolver(9)                        // Solver with magnetoelastic interaction
fixdt = 1e-14                       // Set a fixed time step
autosave(m, 1e-9)                   // Magnetization output
autosave(Edens_mel, 1e-9)           // Magnetoelastic energy density output
autosave(Edens_total, 1e-9)         // Total energy density output
run(400e-9)                         // Running
```

The SH wave and Rayleigh wave are simulated by using a finite element model (FEM). The SH wave is generated as a fundamental mode with the wave propagation direction parallel to the **X** axis of 64°Y-cut $LiNbO_3$ (**x** axis)[4]. The piezoelectric constant matrix is defined by

$$e = \begin{bmatrix} 0 & 0 & 0 & 0 & -2.23 & 3.87 \\ 2.33 & 0.986 & -2.58 & -3.12 & 0 & 0 \\ 0.916 & 0.023 & -2.47 & -1.04 & 0 & 0 \end{bmatrix} \tag{S1}$$

The Rayleigh wave is generated as a fundamental mode with the wave propagation direction perpendicular to the **X** axis of 64°Y-cut $LiNbO_3$ (**y** axis). The piezoelectric constant matrix is transformed to

$$e = \begin{bmatrix} 0.986 & 2.33 & -2.58 & 0 & -3.12 & 0 \\ 0 & 0 & 0 & -2.23 & 0 & 3.87 \\ 0.023 & 0.916 & -2.48 & 0 & -1.04 & 0 \end{bmatrix} \quad (S2)$$

The simulated displacement distributions and admittances of the SH wave and Rayleigh wave are shown in Fig. S2. The mode of vibration for SAW was simulated by using a finite element method (FEM), as shown in Supplementary Fig. S2a, b. Different modes of vibration (Rayleigh wave or SH wave) can be distinguished by analyzing the particle displacement. When the SAW propagation direction is along the **y** axis of the 64°Y-cut $LiNbO_3$ the SAW mode particle displacement consists of the vertical displacement component and the longitudinal displacement component, which indicates a Rayleigh wave mode, as shown in Supplementary Fig. S2b. The simulated resonance frequency of the Rayleigh wave mode is also consistent with that measured in experiments. The SH wave expression in the simulation is defined as follows

$$\boldsymbol{u}_{SH} = \begin{bmatrix} u_x \\ u_y \\ u_z \end{bmatrix} = \begin{bmatrix} 0 \\ A\sin(kx - \omega t) \\ 0 \end{bmatrix} \quad (S3)$$

where A, ω, and t are the amplitude of a SAW, the angular frequency of a SAW, and time, respectively. $k=2\pi/\lambda_{\mathrm{SAW}}$ is the wavenumber of a SAW. λ_{SAW} is the wavelength of a SAW. The Rayleigh wave expression is defined as follows

$$\boldsymbol{u}_{\mathrm{Rayleigh}} = \begin{bmatrix} u_x \\ u_y \\ u_z \end{bmatrix} = \begin{bmatrix} A_1 \sin(kx - \omega t) \\ 0 \\ A_2 \sin(kx - \omega t) \end{bmatrix} \quad (S4)$$

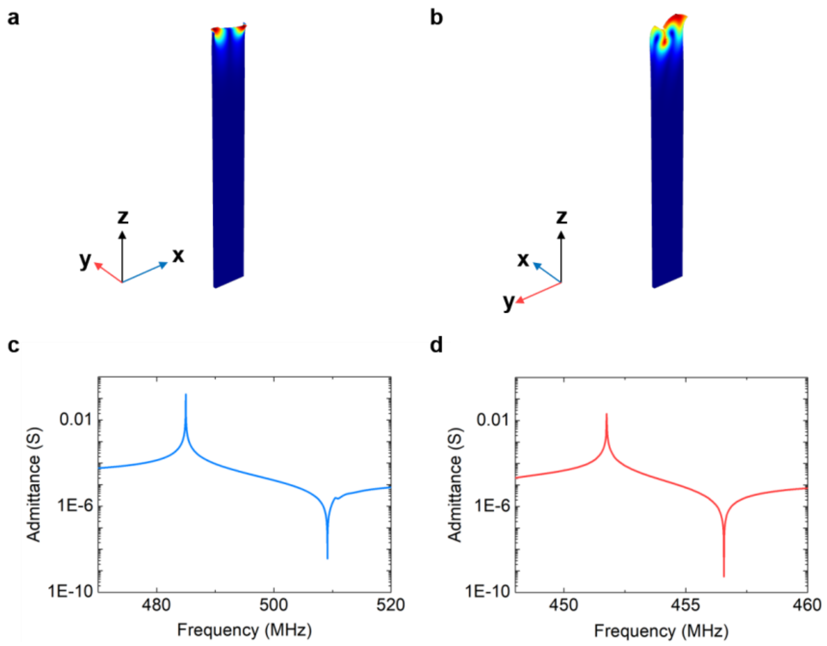

Supplementary Fig. S2 | The simulated displacement distributions and admittances of the SH wave and Rayleigh wave. a, The simulated displacement distribution of the SH wave. **b**, The simulated displacement distribution of the Rayleigh wave. **c,** The simulated admittances of the SH wave. **d**, The simulated admittances of the Rayleigh wave. The wavelength is 8 μm.

The dominant displacement components of Rayleigh waves are different depending on different cut types of piezoelectric substrates[5]. In our FEM simulation, we find that the vertical displacement of the

Rayleigh wave is 1.5 times as large as its longitudinal displacement along **x** when the wave propagation direction is along the **y** axis of 64°Y-cut $LiNbO_3$, as shown in Fig. S3. We have thus mainly considered the vertical displacement of the Rayleigh waves in the micromagnetic simulations as shown in Fig. 1b. We have also analyzed whether the longitudinal displacement along **x** alone or a combination of longitudinal and vertical displacement of Rayleigh wave can move skyrmions. We found that they both move skyrmions much less effectively than the particle displacement along *y* of the SH wave. Fig. S4 illustrates the position changes of skyrmions under the action of a Rayleigh wave and an SH wave, respectively. The moving distance of a skyrmion under a Rayleigh wave is smaller compared to that under an SH wave.

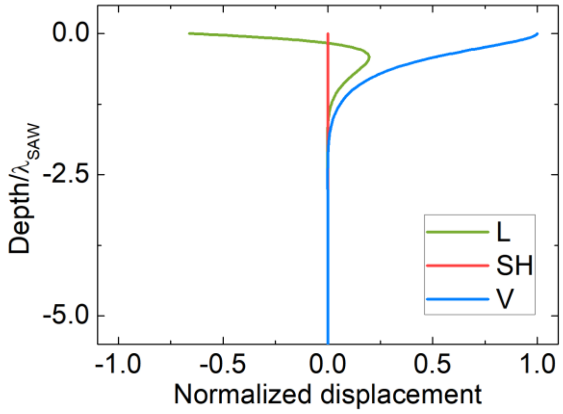

Supplementary Fig. S3 | The simulated particle displacement components of the Rayleigh wave when the wave propagation direction is along the **y** axis of 64°Y-cut $LiNbO_3$. The longitudinal displacement component (L), the shear horizontal displacement component (SH), the vertical displacement component (V).

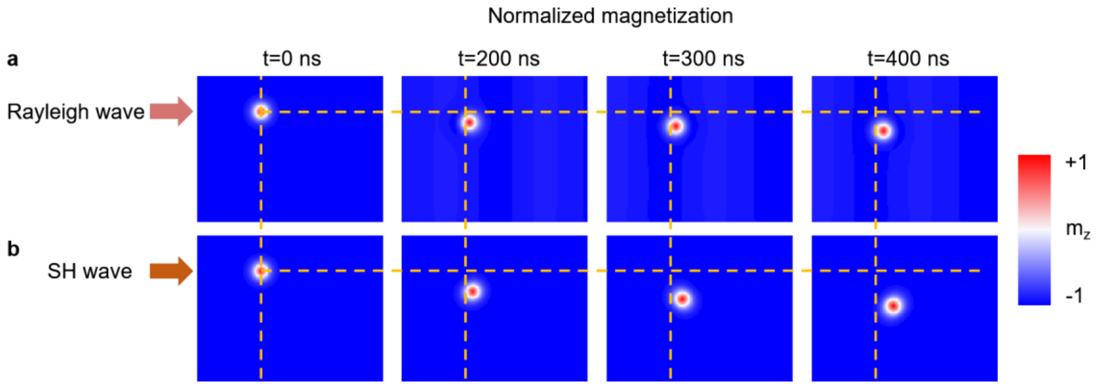

Supplementary Fig. S4 | The simulated position changes of skyrmions under a Rayleigh wave and an SH wave. a, The position changes of a skyrmion under a Rayleigh wave. **b,** The position changes of a skyrmion under an SH wave.

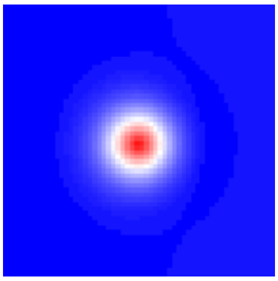

Supplementary Fig. S5 | The simulated skyrmion under an elastic wave with periodic shear vertical displacements.

Supplementary Fig. S6a shows the simulated magnetoelastic force density distribution of a skyrmion with the ratio of the SAW wavelength to the skyrmion diameter $\frac{\lambda}{D_{\mathrm{sk}}} = 800$. In this case, the magnetoelastic force density distribution of the skyrmion is nearly symmetric. And the net magnetoelastic force on the skyrmion can be calculated in the order of 3×10^{-2} nN. By continuously decreasing the wavelength of a SAW (and hence the ratio of $\frac{\lambda}{D_{\mathrm{sk}}}$), the shear strain gradient across the skyrmion becomes non-uniform which induces a larger net effective magnetoelastic force, as shown in Supplementary Fig. S6b.

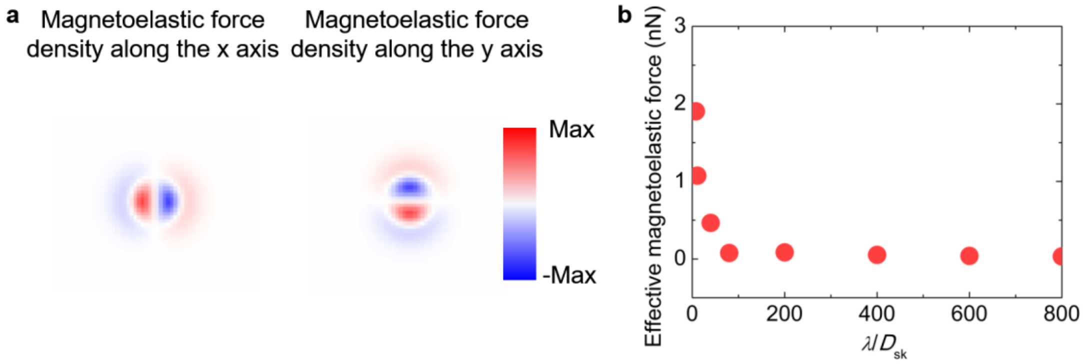

Supplementary Fig. S6 | The simulation of the magnetoelastic force. a, The simulated magnetoelastic force density distribution of a skyrmion under an SH wave when the ratio of the SAW wavelength to the skyrmion diameter $\frac{\lambda}{D_{\mathrm{sk}}} = 800$. **b,** The net effective magnetoelastic force on the skyrmion as a function of $\frac{\lambda}{D_{\mathrm{sk}}}$.

III. Analysis of skyrmions motion

When an SH wave propagates along the **x** axis in thin films, the spatiotemporally varying shear displacement ($u_y = A\sin(kx-\omega t)$) component is dominant for an SH wave, where A, k, ω, and t are the amplitude, the wavenumber, the angular frequency of the SAW, and time, respectively. The shear strain ($\varepsilon_{xy} = kA\cos(kx-\omega t)$) component of an SH wave is dominant over the others. In the case of considering only the shear strain ε_{xy}, spatially inhomogeneous in-plane magnetoelastic effective fields are generated in the x and y axes. The SH wave with a wavelength of 10 μm also moves skyrmions, as shown in Fig. S7.

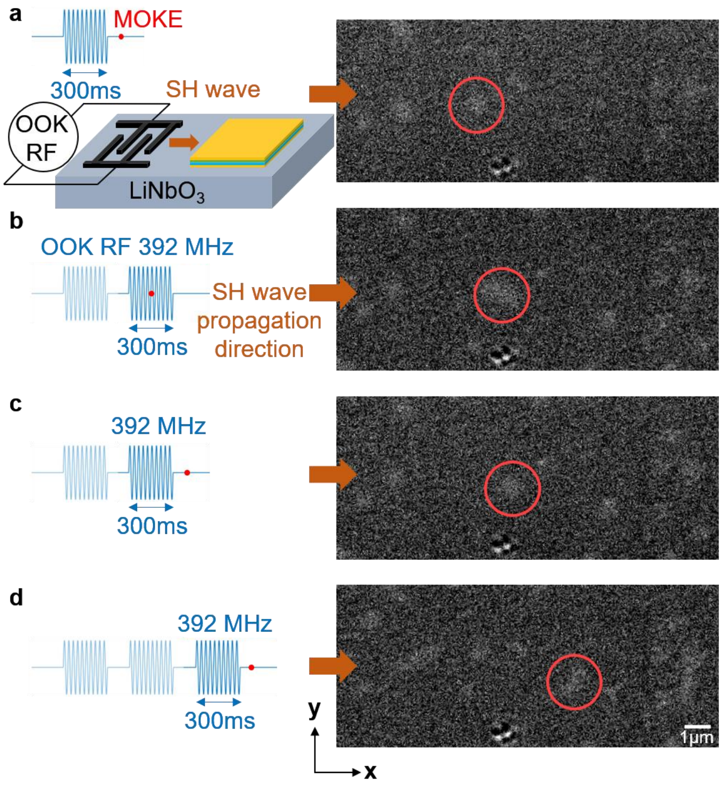

Supplementary Fig. S7 | The skyrmion motion driven by SH waves with the wavelength of 10 μm. a, The creation of magnetic skyrmions by exciting a propagating SH wave pulse (λ_{SAW} = 10 μm) with a duration of 300 ms. **b**, The motion of a magnetic skyrmion during the propagating SH wave. **c**, The MOKE image after exciting an SH wave pulse with a duration of 300 ms. **d**, The MOKE image after exciting an SH wave pulse with a duration of 300 ms once again. The wavelength is 10 μm. The RF power is 26.6 dBm. The scale bar is 1 μm.

We also discuss the factors influencing the skyrmion motion distances. The effect of the wavelength on the motion distances of skyrmions is investigated in experiments, as shown in Fig. S8a. The Rayleigh wave does not drive the motion of skyrmions in experiments. We focus on the skyrmions with the motion distances over 1 μm. Statistically, 32% of skyrmion population shows motion distance d>1 μm when the wavelength of the SH wave is 8 μm. 17.2% of skyrmion population shows motion distance d>1 μm when the wavelength increases to 10 μm. The reduction in a wavelength from 10 μm to 8 μm produces enhanced strains of SH waves, results in more skyrmion numbers in improved motion distances. Most of the skyrmions moving less than 1 μm indicate creep motions in a low-depinning regime and are easily trapped by pinning sites. The effect of exciting RF power that controls wave amplitude on the motion distances of skyrmions is also investigated. The amplitude of the strain generated by a SAW is proportional to the RF power before the saturation power of SAW. The number of moving skyrmions reduces when the RF power decreases from 26.6 dBm to 20 dBm, as shown in Fig. S8b. Increasing RF powers and decreasing the wavelength are effective methods for depinning, together with an increased motion distance.

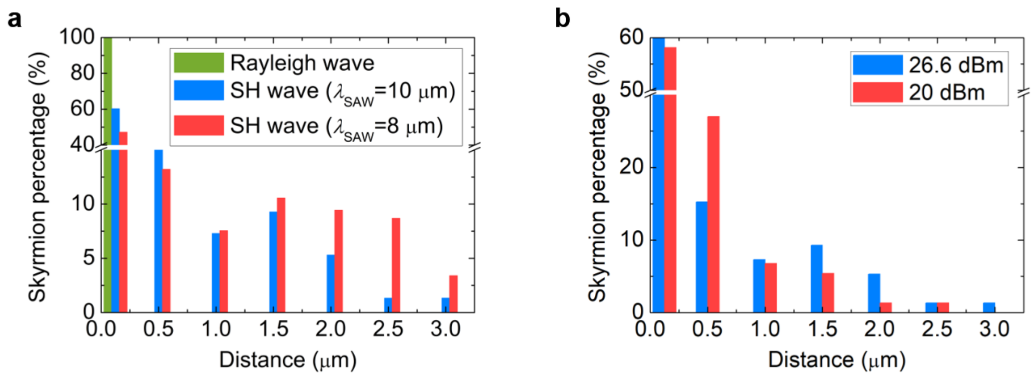

Supplementary Fig. S8 | Statistical analysis of skyrmions motion distances. a, The measured skyrmions motion distances driven by SH waves with different wavelengths. **b,** The relationship between skyrmions motion distances and RF powers.

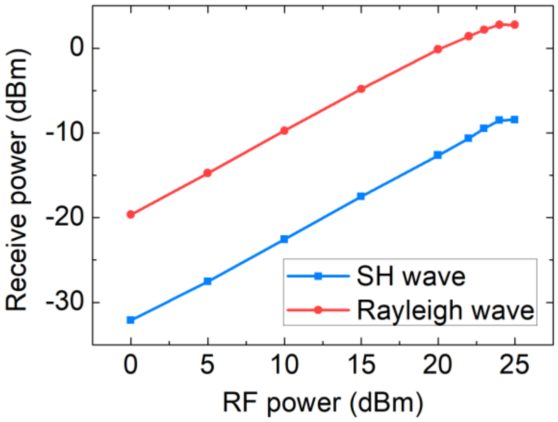

Supplementary Fig. S9 | The received power of the receiving IDTs as a function of RF power for the SH wave and Rayleigh wave.

The skyrmion motion driven by the SAW can be described by a modified Thiele equation[6]:

$$\mathbf{G}\times\mathbf{V}-\alpha\mathbf{D}\mathbf{V}+\mathbf{F}=0 \tag{S5}$$

where $\mathbf{G} = (0, 0, -4\pi Q)$ is the gyromagnetic coupling vector. Q is the topological charge. α is the Gilbert damping parameter. $\mathbf{D}$ is the dissipative force tensor. $\mathbf{V} = (V_x, V_y)$ is the skyrmion velocity along the **x** and **y** axes. $\mathbf{F} = (F_x, F_y)$ is the effective magnetoelastic forces along the **x** and **y** axes. The effective forces induced by an SH wave on the skyrmion in a magnetic thin film with an isotropy magnetostriction coefficient are defined by[7-10]

$$F_x = A_{xx}\nabla\varepsilon_{xx} + A_{xy}\nabla\varepsilon_{xy} + A_{xz}\nabla\varepsilon_{xz} + A_{yx}\nabla\varepsilon_{yx} + A_{zx}\nabla\varepsilon_{zx} \tag{S6}$$

$$F_y = A_{yy}\nabla\varepsilon_{yy} + A_{yx}\nabla\varepsilon_{yx} + A_{yz}\nabla\varepsilon_{yz} + A_{xy}\nabla\varepsilon_{xy} + A_{zy}\nabla\varepsilon_{zy} \tag{S7}$$

where A_{ii} is a coefficient depending on the spin texture of a skyrmion[7,10]. A_{xx} and A_{yy} depend on the first order magnetoelastic coupling constant B_1. A_{xy}, A_{xz}, A_{zx}, A_{yx}, A_{yz}, and A_{zy} depend on the second order magnetoelastic coupling constant B_2[7,10]. ε_{ii} is a strain component induced by a SAW. F_x and F_y are proportional to the strain gradients and the magnetostriction coefficient. According to Eq. S5, the skyrmion velocity is defined by

$$V_x = \frac{\alpha D F_x - G F_y}{\alpha^2 D^2 + G^2}$$
$$V_y = \frac{G F_x + \alpha D F_y}{\alpha^2 D^2 + G^2} \qquad \text{(S8)}$$

It can be found that the skyrmion velocity is related to the Gilbert damping parameter. From Eq. S8, the skyrmion deflection angle is defined by

$$\phi_{sk} = \arctan\left(\frac{G + \alpha D \frac{F_y}{F_x}}{\alpha D - G \frac{F_y}{F_x}} \right) \qquad \text{(S9)}$$

For magnetic thin films with an isotropy magnetoelastic coupling constant, the magnetostriction coefficient would be nullified in the equation for skyrmion deflection angle. In our simulations, we also tested using various magnetoelastic coupling constants but observed no change in the skyrmion deflection angle.

Fig. S10 shows the deflection angle as a function of damping parameters with different F_y/F_x. An increased damping parameter from 0.01 to 0.07 results in a reduced deflection angle. The deflection angle of the skyrmion motion driven by an SH wave is also influenced by F_y/F_x. F_x and F_y are proportional to the shear strain gradient. They are nonzero for the SH wave, and therefore also influence the deflection angle.

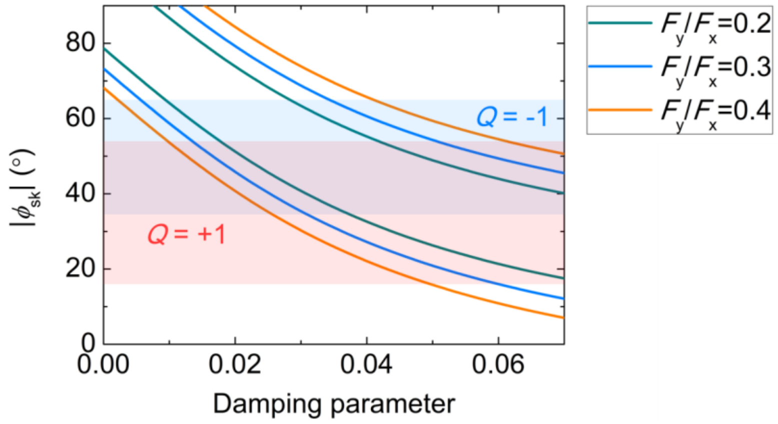

Supplementary Fig. S10 | The experimental (blue and red regions) and numerically calculated (curves) skyrmion deflection angles (ϕ_{sk}) versus damping parameters with different F_y/F_x.

When the RF power is applied to IDTs, local heat can be generated in the magnetic thin film, resulting in the establishment of a thermal gradient[11]. We quantified the amplitude of the thermal gradient in the sample induced by the RF power using a time-resolved thermography camera[12], as shown in Fig. S11. In our sample, the distance from the edge of the magnetic film to the edge of the IDT is 62 μm. Temperature images were captured after exciting SAW with a pulse duration of 300 ms and an RF power of 26 dBm at room temperature, which are the standard experimental parameters for skyrmion generation and motion. Our finding reveal that the temperature rise in the magnetic film is less than 1 °C, and therefore would have minimal impact on the magnetic properties of the thin film. The generated thermal gradient in the

magnetic film is measured to be 1.2 mK/μm, indicating that the thermal gradient induced by IDTs is negligible in influencing the skyrmion generation and motion[13].

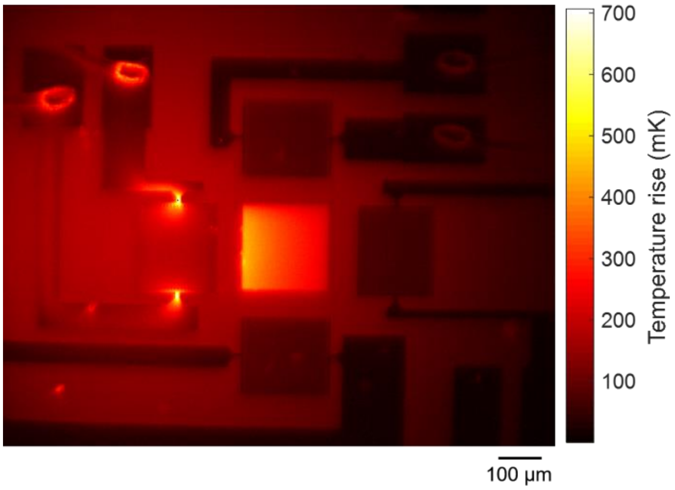

Supplementary Fig. S11 | The temperature rise image of the device at the room temperature when a 300 ms RF pulse with the power of 26 dBm are applied to the IDT. The scale bar is 100 μm.

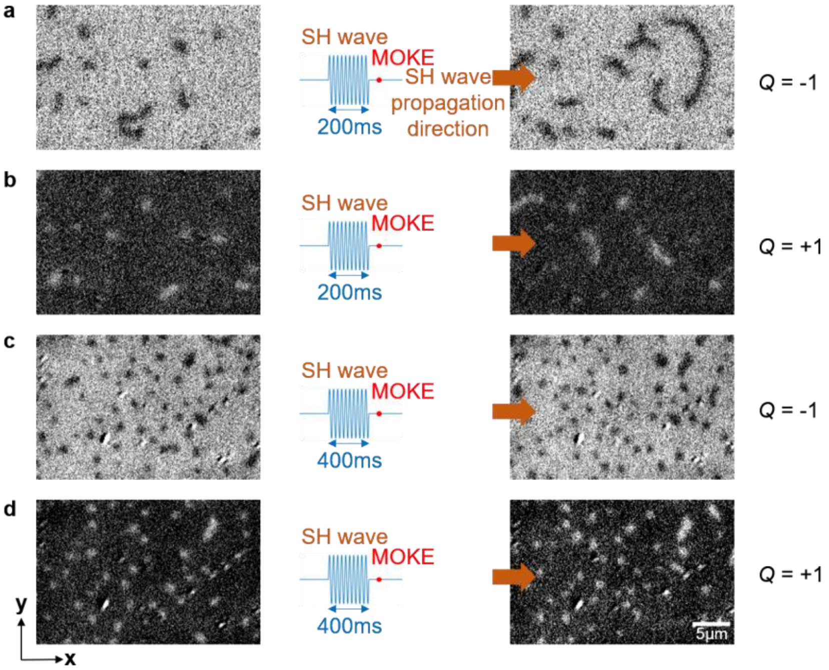

Supplementary Fig. S12 | Evolution of magnetic textures by applying SH waves. a, MOKE images of Q = -1 skyrmions after exciting 200 ms SH waves. **b**, MOKE images of Q = +1 skyrmions after exciting 200 ms SH waves. **c**, MOKE images of Q = -1 skyrmions after exciting 400 ms SH waves. **d**, MOKE images of Q = +1 skyrmions after exciting 400 ms SH waves. The wavelength is 8 μm. The RF power is 26 dBm. The scale bar is 5 μm.

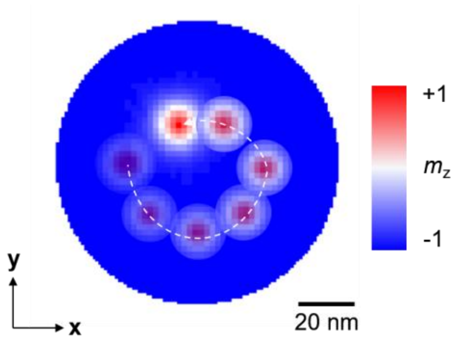

Supplementary Fig. S13 | The micromagnetic simulation for the motion of a skyrmion driven by an SH wave in a circular magnetic film[14]. The wavelength is 8 μm. The scale bar is 20 nm.

IV. Analysis of the effective power density to drive skyrmion motion

The effective power density for a single skyrmion driven by a SAW is defined by

$$P = \frac{\frac{1}{L}\int_0^L (P_{\mathrm{SAW}} - \beta x)dx}{W \bullet L} \tag{S10}$$

where P_{SAW} is the applied RF power, β is the SAW propagation loss, W is the width of the magnetic film, and L is the length of the magnetic film. The effective power density for a single skyrmion with a speed of around 10 μm/s is 74.6 W/cm^2 when the applied RF power is 26 dBm. Table. S1 lists the performance of our work compared with other driven methods.

Supplementary Table. S1 Comparison of different skyrmion driven methods

Skyrmion Driven method	Mechanism	Power density	Active skyrmion area	Local heating	Material system	Reference
Current	Spin-orbit torques (SOT)	4.4×10^3~ 2.26×10^4 W/cm^2	10 μm×50 μm	Yes	YIG/TmIG/Pt	[15]
Current	SOTs	1.5×10^4 W/cm^2	2 μm×5 μm	Yes	$[Pt/Co/Ta]_{15}$	[16]
Thermal gradient	Repulsive and entropic forces, thermal SOTs	425 W/cm^2	4 μm×30 μm	Yes	$[Ta/CoFeB/MgO]_{15}$	[17]
Surface acoustic waves	Magnon-phonon interaction	74.6 W/cm^2	200 μm×200 μm	No	Ta/CoFeB/MgO/Ta	This work

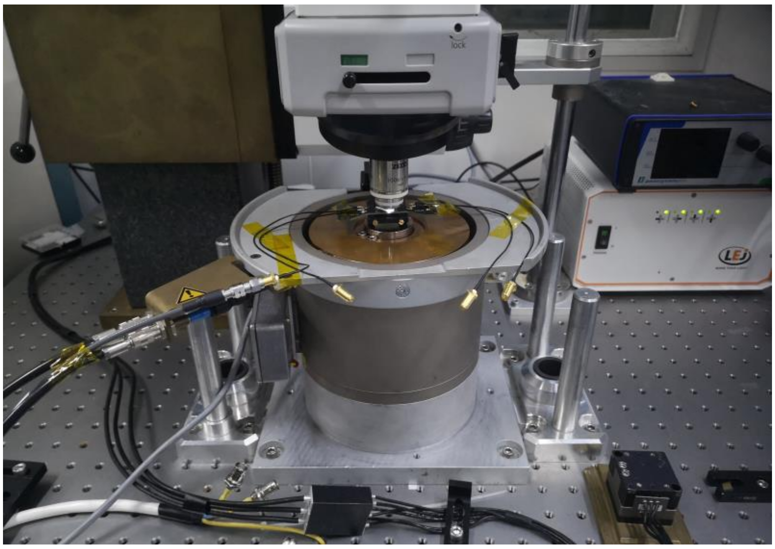

Supplementary Fig. S14 | The setups of the MOKE microscope for SAWs-driven skyrmion experiments.

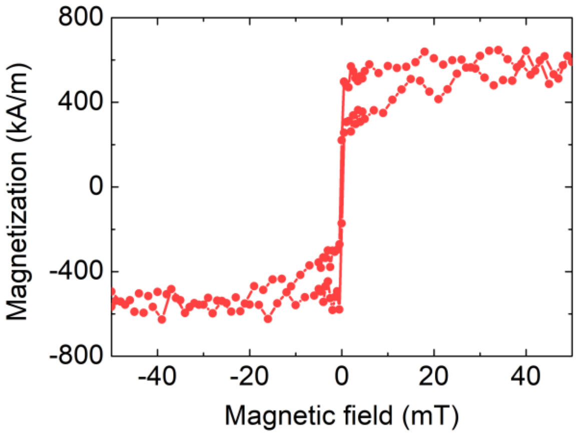

Supplementary Fig. S15 | The measured out-of-plane magnetic hysteresis loop of Ta/$Co_{20}Fe_{60}B_{20}$/MgO/Ta by a vibrating sample magnetometer.

Supplementary Movie 1:

This movie contains the whole set of images shown in Fig. 3a-d, in which Q = +1 skyrmion motion after exciting SH wave pulses.

Supplementary Movie 2:

This movie contains the whole set of images shown in Fig. 3e-h, in which Q = -1 skyrmion motion after exciting SH wave pulses.

References

1. Vansteenkiste, A., Leliaert, J., Dvornik, M., Helsen, M., Garcia-Sanchez, F. & Waeyenberge, B. V. The design and verification of MuMax3, *AIP Adv.* **4**, 107133 (2014).

2. Mulkers, J., Waeyenberge, B. Van, Milošević, M. V. Effects of spatially engineered Dzyaloshinskii-Moriya interaction in ferromagnetic films. *Phys. Rev. B* **95**, 144401 (2017).
3. Vanderveken, F., Mulkers, J., Leliaert, J. *et al*. Finite difference magnetoelastic simulator. *Open Research Europe* **1**, 1-23 (2021).
4. Mandal, D., Banerjee, S. Surface Acoustic Wave (SAW) Sensors: Physics, Materials, and Applications. *Sensors*, **22**, 820 (2022).
5. Yamanouchi, K., Shibayama, K. Propagation and Amplification of Rayleigh Waves and Piezoelectric Leaky Surface Waves in $LiNbO_3$. *J. Appl. Phys.* **43**, 856–862 (1972).
6. Thiele, A. A. Steady-state motion of magnetic domains. *Physical Review Letters*, **30**, 230-233 (1973).
7. Nepal, R., Güngördü, U., Kovalev, A. A. Magnetic skyrmion bubble motion driven by surface acoustic waves. *Appl. Phys. Lett.* **112**, 112404 (2018).
8. Yanes, R., Garcia-Sanchez, F., Luis, R. F. *et al*. Skyrmion motion induced by voltage-controlled in-plane strain gradients. *Appl. Phys. Lett.* **115**, 132401 (2019).
9. Vanderveken, F., Mulkers, J., Leliaert, J. *et al*. Confined magnetoelastic waves in thin waveguides. *Phys. Rev. B* **103**, 054439 (2021).
10. Chen. C., Wei, D., Sun, L., Lei N. Suppression of skyrmion Hall effect via standing surface acoustic waves in hybrid ferroelectric/ferromagnetic heterostructures. *J. Appl. Phys.* **133**, 203904 (2023).
11. Chen, C., Fu, S., Han, L., *et al*. Energy Harvest in Ferromagnet-Embedded Surface Acoustic Wave Devices. *Adv. Electron. Mater.* **8**, 2200593 (2022).
12. O. Breitenstein, W. Warta, and M. Langenkamp, *Lock-in Thermography: Basics and Use for Evaluating Electronic Devices and Materials* (Springer, Berlin/Heidelberg, 2010).
13. Wang, Z., Guo, M., Zhou, HA. *et al*. Thermal generation, manipulation and thermoelectric detection of skyrmion. *Nat. Electron.* **3**, 672-679 (2020).
14. Yang, Y., Ji, Y., Zhang, C., & Nan, T. Magnetic skyrmion dynamics induced by surface acoustic waves. *J. Phys. D: Appl. Phys.* **56**, 084002 (2023).
15. Vélez, S., Ruiz-Gómez, S., Schaab, J. *et al.* Current-driven dynamics and ratchet effect of skyrmion bubbles in a ferrimagnetic insulator. *Nat. Nanotechnol.* **17**, 834–841 (2022).
16. Woo, S., Litzius, K., Krüger, B. *et al.* Observation of room-temperature magnetic skyrmions and their current-driven dynamics in ultrathin metallic ferromagnets. *Nat. Mater.* **15**, 501–506 (2016).
17. Wang, Z., Guo, M., Zhou, HA. *et al.* Thermal generation, manipulation and thermoelectric detection of skyrmions. *Nat. Electron.* **3**, 672–679 (2020).
