# Supplementary material for: Acoustic-driven magnetic skyrmion motion
Source: Nat Commun. 2024 Feb 3;15:1018. doi: 10.1038/s41467-024-45316-w (PMC10838300; doi:10.1038/s41467-024-45316-w)
Supplement: Supplementary file 2 — Description of Additional Supplementary Files [file 41467_2024_45316_MOESM2_ESM.pdf]

Title: Supplementary Movie 1:
Description: This movie contains the whole set of images shown in Fig. 3a-d, in which Q = +1 skyrmion motion after exciting SH wave pulses.

Title: Supplementary Movie 2:
Description: This movie contains the whole set of images shown in Fig. 3e-h, in which Q = -1 skyrmion motion after exciting SH wave pulses.
